# Use of Simple Telemetry to Reduce the Health Impacts of Fuel Poverty and Living in Cold Homes

**DOI:** 10.3390/ijerph16162853

**Published:** 2019-08-09

**Authors:** Adam Pollard, Tim Jones, Stephen Sherratt, Richard A. Sharpe

**Affiliations:** 1 Numeration Systems, Ltd., Health and Well-Being Innovation Centre, Truro TR1 3FF, UK; 2 Community Energy Plus, Truro TR1 2SJ, UK; 3 Health and Well-being and Public Health, Cornwall Council, Truro TR1 3AY, UK; 4 European Centre for Environment and Human Health, University of Exeter Medical School, Truro TR1 3HD, UK

**Keywords:** fuel poverty, intervention, indoor temperature, public health

## Abstract

Introduction: In Great Britain, roughly half of people with at least one long-standing illness (LSI) live in low-income households. Lower-income households are at risk of fuel poverty and living in a colder house, which can worsen certain health conditions, causing related morbidity and mortality. This pilot study aimed to assess whether raising occupants’ awareness of indoor temperatures in the home could initiate improved health and well-being among such vulnerable residents. Methods: Thermometers were placed inside a manufactured bamboo brooch to be worn or placed within homes during the winter of 2016/17. These devices were supplied to households (n = 34) already assisted by Community Energy Plus, which is a private social enterprise in Cornwall, United Kingdom (UK), using initiatives aimed at maintaining “healthy homes”. Questionnaires were supplied to households before devices were supplied, and then again at the end of a three-month period, with further questions asked when devices were collected. Temperatures were recorded automatically every half-hour and used to draw inference from questionnaire responses, particularly around health and well-being. Results: Questionnaires were completed by 22 households. Throughout the winter, those declaring the poorest health when supplied with devices maintained homes at a higher average temperature. There were also indications that those with raised awareness of interior temperatures sought fewer casual medicines. Conclusion: Simple telemetry could play a role in the management of chronic health conditions in winter, helping healthcare systems become more sustainable. The need for higher indoor temperatures among people with an LSI highlights the need to consider this approach alongside more sustainable household energy-efficiency improvements. A larger study is needed to explore this further and quantify the cost benefit of this approach.

## 1. Introduction

Approximately 36% of people in Great Britain have at least one long-standing illness or disability (LSI), of whom just over half state their condition limits day-to-day activities [1]. Among people with an income of less than £10,000 GBP per year ($13,000 USD, the lowest income group reported), 45% report an LSI, with two-thirds of these reporting their day-to-day activities as limiting [1]. These lower-income populations are also more likely to live in poor housing conditions and suffer from the effects of fuel poverty (i.e., those unable to adequately heat the home due to the cost of fuel and/or energy efficiency of the home [2]), which increases the risk of cold-related morbidity and mortality [3,4,5,6]. While home energy efficiency interventions can deliver positive health outcomes (i.e., by making homes more affordable to heat) [7], some have been found to exacerbate some conditions such as increasing the risk of asthma [8]. This can result from reduced indoor air quality and resident awareness of the importance in maintaining adequate ventilation and indoor temperatures [8,9]. This means that there is conflicting evidence on which interventions work to lower the burden of cold homes on society and healthcare systems [10].

Identifying sustainable interventions that can help raise awareness and deliver improvements in the way people heat their home could help alleviate the impacts of cold homes on health and particular in the management of LSIs. Suitable approaches are yet to be fully explored, which is the focus of this study. This is of public health interest, because fuel poverty poses a significant public health and socioeconomic burden worldwide [10]. For example, living in cold and damp homes in the United States is thought to cost over $3.5 billion in the management and treatment of asthma [11]. When including healthcare costs, lost taxes, and welfare payments, poor housing is thought to cost between £36 billion to £40 billion in the United Kingdom (UK) [12]. Furthermore, the demand for National Health Service (NHS) treatment in the UK is increasing due in to an ageing population. This is a concern to healthcare providers, because increased cold-related morbidity and mortality [13,14] often means that hospitals are frequently at or near capacity, with access to emergency treatment being an issue [15]. Interventions raising the thermal comfort of homes have the potential to reduce the impact of these seasonal risks on health. In the UK, this approach has the potential to save (the NHS) £848 million/year, with a £435 million/year savings resulting from reducing falls in the home [12]. 

Fuel poverty has become an emergent topic over the last decade [2] because it is thought to affect between 6–34% of homes [4]. Patterns of fuel poverty vary both spatially within countries and socially between different groups [16,17,18]. In England, 10.6% of households were thought to be living in fuel poverty in 2016, which can increase (14.9%) in more rural areas such as Cornwall in the southwest (SW) of England [19]. 

Living in cold homes accentuates both a physiological and psychological effect when the vulnerable are exposed to adverse cold temperatures within the home [20], and poses a significant risk to residents’ physical and mental well-being [2]. Around 40% of the excess winter deaths in the UK attributed to cold homes were from cardiovascular diseases, and 35% were from respiratory disease [13]. Also, for every 1 °C drop in temperature below 5 °C, general practitioner (GP) consultations for respiratory illness in older people increase by 19%, and a 1 °C drop in living-room temperature results in a rise in blood pressure amongst those aged between 65–74 [21], which increases the risk of falling. Additionally, fuel-poor households may choose to only heat one room within the property (to save money), which further increases the risk of falling. This can occur as a result of blood pressure changes when moving from a warm to a cold room in the property [22].

Fuel poverty is a complex and pervasive policy problem to resolve, largely because of the difficulty in identifying and targeting associated households [23]. Alleviating the risk of fuel poverty, e.g., through energy efficiency and behavioural measures, [10] and the better management of LSIs through earlier intervention and preventative actions, offers an opportunity to reduce the burden on society and cut the resources consumed and emissions generated by health systems. Future housing interventions need to avoid a range of potential unintended consequences resulting from household energy efficiency measures [9]. These need to take a more holistic approach that considers the ‘whole dwelling’ and adequate ventilation and heating [2]. 

The recommended indoor temperature for a healthy indoor environment lies between 18–26 °C [24]. While the evidence base for this is questionable [22], higher indoor temperatures maintained during winter can result in a range of health improvements, particularly among the most vulnerable populations [7]. 

Through the use of individual temperature monitors, we aimed to assess whether continuous monitoring helps residents maintain healthier indoor temperatures (i.e. to reduce the risk of living in cold homes [25]) and if this could contribute to health improvements. Temperature loggers have been used previously to investigate the health benefits associated with government home improvement initiatives in the UK [26]. In this pilot study, we used these loggers to inform future service delivery and assess how this could affect the use of pharmacists and NHS services. This approach has been taken to test the feasibility of using data loggers to inform future fuel poverty interventions.

## 2. Materials and Methods

This cross-sectional descriptive pilot study was implemented by Community Energy Plus (CEP). Due to the nature of this pilot, the study was conducted as a service improvement to inform CEP’s fuel poverty support and services (i.e., following a quality improvement framework [27]). For this reason, all the participants provided formal written consent on behalf of the household, which included responses to the questionnaires and the recording of indoor temperatures. The consenting process was undertaken by trained CEP staff who were accompanied by a family member where appropriate, which in this case included one participant who had mild dementia. The pilot study was conducted with households across Cornwall, which has the high levels of fuel poverty. This is exacerbated by lower average earnings and a high proportion of households (42%) who remain disconnected to the mains gas supplies, which provide a cheaper form of fuel. Consequently, these homes are forced to rely on more expensive forms of energy [28]. Therefore, those living in more rural areas are more vulnerable to the impacts of energy price increases, particularly those living in the private rental sector [29,30] and suffering from one or more LSI. 

To assess the potential for individual temperature monitors, a total of 34 households who were in contact with Community Energy Plus (CEP) and had one or more LSIs were asked to participate in this pilot study. CEP provides a range of support services to alleviate the pressures of fuel poverty across Cornwall. These include a telephone-based helpline and enabling minor home improvements such as insulation and heating-system improvements aimed at maintaining a healthy, dry home through helping residents acquire necessary funding. This social enterprise has been working with vulnerable people in Cornwall to assist them in achieving and maintaining adequate levels of thermal comfort in their homes. Households that struggle with the costs of keeping their home warm (e.g., due to the cost of fuel and energy efficiency of a property) are described as “fuel poor”. There are a number of challenges associated with identifying and quantifying the extent of fuel poverty. Different approaches used to define fuel poverty have been widely contested in the UK. Notably, as a result of the Hills Review, the Government changed the definition use for fuel poverty in England. This changed from the inclusion of households who spend 10% of their disposable income on energy, to the Low Income High Cost (LIHC) criteria [31,32,33,34]. It is important to note that this is not a term that these householders would use to describe themselves. 

To assess whether wearable telemetry (i.e., a thermometer with a low-temperature alarm) has the potential to raise awareness of the effects of cold homes among those living in fuel poverty, 34 wearable or freestanding temperature loggers were distributed to 34 homes during three months of the winter of 2016/17. The loggers were equipped with sensors that triggered a flashing light-emitting diode (LED) if the temperature in the immediate vicinity fell below 18 °C or rose above 26 °C. This provided participants and their families with an alert for changes in temperature that could exacerbate their LSI. Temperatures were recorded every 15 minutes with the number of readings below 18 °C and 15 °C noted. The loggers fed recorded temperatures into comma-separated value (CSV) tabulations in which the logger’s serial number was recorded alongside the date and time of measurement. Then, the CSV files were imported into Microsoft Excel where temperature data was analysed using in-built functionality.

One of CEP’s qualified energy advisors visited households and delivered the temperature loggers. The advisors assessed participants’ individual situation and needs, and provided basic guidance on what actions they could take in the event of receiving a low or high temperature alert. This generally involved simple behavioural change such as using the alert as a reminder to switch central heating systems on, turning up the system thermostat, closing windows, making a hot drink, wearing more clothing layers, or simply trying to be more active. The home visits also provided in-depth energy efficiency assessments and, where appropriate, referrals for measures including draught excluders, insulation, or equipment to reduce condensation and dampness. 

Questionnaires were designed using a closed questioning technique. They were designed to collect data on participants’ households. Structured baseline and follow-up questionnaires were conducted with an adult aged over 18 years within each household to assess the effectiveness of the intervention. These aimed to determine any change in participants’ health and well-being. Answers to these questions comprised fixed categories, allowing them to be set in context against the ambient temperatures to which respondents were exposed. Questions included, for example:

1. How would participants rate their health at the project baseline compared with the previous year?
2. Is there is a permanent household member with an LSI that is known to be worsened by the cold?
3. Have there been any changes to health and well-being in the previous three months?
4. Which health problems had they experienced in the previous three months?
5. Which health care services have been used in the last three months for the LSI?
6. Since engaging with CEP for energy efficiency advice, have there been any changes to health and well-being?
7. What services have been sought in the last three months for any respiratory, cardiovascular, circulatory, or mental health conditions or for cold/flu?
8. Has health and quality of life improved as a result of the CEP intervention?
9. If so, how much has the CEP intervention helped?

At the end of the winter, a second interview was conducted to establish whether the loggers helped with the management of LSIs through the winter by raising the awareness of ambient temperature to which participants were exposed. The following questions were asked:

1. Were participants more aware of the temperature in the house?
2. Did they feel less vulnerable, anxious, or stressed this winter compared to last?
3. Did they feel as cold this winter?
4. Had they been feeling poorly this winter, and if so, how did it compare to last winter?
5. Had they visited the local chemist to speak to the pharmacist or buy casual medicines to relieve them of certain conditions?
6. Compared to last year, had participants used healthcare services less frequently?
7. Did they feel the temperature loggers had helped?

Temperature data captured by each of the loggers were analysed and grouped according to answers provided by participants to each of these questions. Then, basic differential analysis was performed on questionnaire answers as part of an intervention to raise awareness. 

Small sample sizes within this pilot study prevented us from using formal statistical analysis. However, we present a basic analysis with some questionnaire answers grouped according to response alongside measures of temperature, which are presented for contextual presentation. Bias within the questionnaire responses was analysed through use of the chi-squared test with the null hypothesis being a uniform distribution of responses throughout the categories or the difference between positive and negative responses. Between-category variation from uniformity is presented by an overall *p*-value per question. 

## 3. Results

Out of 34 households willing to be supplied with thermometers, 22 households participated in this study through completing questionnaires (representing a response rate of 65%). The majority of participants completing the questionnaire (73%) were aged over 65 years of age, seven of whom described themselves as elderly and living alone. Half of respondents were suffering from respiratory illness, and approximately 30% were living without adequate heating or ventilation. Seven participants reported a cardiovascular condition, while mental illness was cited by three respondents, and a single case of dementia was also noted.

Throughout the trial, the thermometers recorded temperatures that fell between 9–41 °C with a mean of 19.3 °C. The temperature throughout each day reached an average low point of 16.6 °C between 04:00–05:00, whilst the highest temperatures each day were reached between 18:00–19:00 (21.6 °C). The average temperature recorded was higher within each group with a given LSI than without that condition. A small difference in average temperature was recorded for those with respiratory illnesses: the average temperature recorded during winter 2016/17 was 0.7 °C higher than those without such an illness. 

Three respondents described their health as good, with 13 describing their health as poor. In the year previous to the intervention, all but five participants had noticed deterioration in their health, with half of all participants stating that their health had become much worse. The average temperature recorded by participants increased as stated health worsened, with a differential of 1.6 °C between homes containing participants describing their health as “good” versus those with “poor” health. A similar rise in average temperature recorded (1.5 °C) occurred between groups of participants stating their health was “the same” as 12 months previously versus those describing their health as “much worse”.

At least half of participants had noticed a deterioration in their well-being in the previous three months prior to the intervention for each dimension listed in Table 1 (all *p* < 0.05). The most marked deterioration was expressed in terms of participants feeling anxious (*p* = 0.001), relaxed, having emotional well-being (both *p* = 0.002), and feeling comfortable (*p* = 0.003). Over the same period, the most common issues among participants were cardiovascular and circulatory, with four participants noting severe circulatory problems (Table 2). The number of patients not experiencing symptoms of cold/flu or learning disabilities was statistically significantly lower (both *p* < 0.01) than those experiencing symptoms.

Discounting the respondent with “better” health, the average maintained temperature increased with deterioration in health and well-being (Table 1), with the largest temperature differentials between those experiencing severe illnesses against those without that illness for each LSI (Table 2). Those with severe mental illness recorded average temperatures that were 2.9 °C higher than those without a mental illness, whilst the same differential for those with circulatory problems was 1.6 °C.

### 3.1. Impact on Health, Well-Being, and Local Services

The dependency of the cohort on local healthcare services during the three months prior to deployment of thermometers was higher in those who maintained their homes at higher temperatures. Participants averaged one monthly GP visit and almost two hospital visits over the period (a total 30 hospital outpatient appointments and eight Accident and Emergency Department (A & E) attendances).

The average temperature was 0.9 °C higher in the homes of participants visiting the GP on at least four occasions during the previous three months versus those who did not. Among those requiring a hospital appointment, homes were maintained at least 1 °C higher on average compared with those who did not need such appointments.

Since receiving assistance from CEP, participants were split by whether they felt better or worse in terms of physical health, comfort, anxiety, feeling relaxed, quality of life, and emotional well-being (all *p* > 0.05, Table 3). In general, the most common category describing the impact on participants’ stated health and well-being is that of no change. There seems to be very little temperature differential between those expressing their health and well-being as previously improving as opposed to otherwise (Table 3).

Across the three-month period prior to the deployment of badges, participants used NHS services for ailments common in winter on a total of 84 times (44 hospital visits, two A & E attendances, and 38 GP appointments): almost four per participant (Table 4). Participants were significantly more likely to have used a GP across the period (*p* = 0.016) and having not used an Accident and Emergency Department (A & E, *p* < 0.001).

For winter illnesses, those requiring GP or A & E treatment in the three months leading up to the trial of the thermometers experienced colder houses during the trial period (Table 4). Moreover, they experienced more time exposed to temperatures of less than 18 °C or less than 15 °C than those who didn’t require such services. These low temperatures have also been recorded in other studies investigating the impact of indoor environments. Previous studies recorded indoor temperatures ranging from 12.5 to 28.3 °C [13,35].

Nine participants attributed an improved quality of life to the CEP fuel poverty intervention, with 13 people describing it as helping them with their everyday living; five of them stated that it made a big difference. The nine participants expressing an improvement in quality of life recorded temperatures slightly cooler than those who had stated the intervention had not led to such improvements. Furthermore, these nine participants had experienced temperatures of 15 °C or less for 16.3% of the time during the trial, which was up almost 7% on those who had expressed no change in quality of life as a result of the intervention.

### 3.2. Follow-Up Questionnaires

During the follow-up interviews, 16 participants stated that they were more aware of the temperature in the house (*p* = 0.016, Table 5). Of these, the time they were exposed to 15° or less was 14.1% versus 24.7% for those whose felt they were not more aware. 

Eleven respondents felt less vulnerable across the same period, all of whom were more aware of the temperature inside their homes. Four felt at least as vulnerable, all of whom were more aware of the temperatures. There were five participants who did not know if they were more aware of the temperatures and did not know if they felt more vulnerable. Eight participants felt not as cold. For those feeling less vulnerable, they were exposed to 15 °C or less 11.4% of the time, and to 18 °C or less 49.4% of the time; both of these values were lower than for those who felt at least as vulnerable (Table 5). Despite stating they felt colder this winter, nine participants were exposed to 15 °C or less for 13.1% of the time, versus 17.2% of the time for the eight who felt not as cold. 

Of the 21 respondents who responded to the question whether they had felt better than the previous winter, five stated that they did, whilst nine said that they felt no better. Although there was no real recorded difference in the average temperature between these three categories, there were marginal differences in the time that the participants in these categories were exposed to temperatures of 15 °C or less or 18 °C or less (Table 5).

#### 3.2.1. Advice from Pharmacist

Advice from the pharmacist or casual medicines were sought most often by participants with issues relating to breathing, coughing, colds, and sore throats or with issues regarding heart and circulation (Table 6). Visits to the chemist by participants in each of these two categories numbered slightly larger than that required for pain management and musculoskeletal pain.

Those seeking relief via casual medicines or a pharmacist for respiratory and rhinitis-type symptoms or for influenza maintained their household temperatures above 15 °C for a greater proportion of time than those who did not. For those with skin irritations and diarrhoea and vomiting seeking similar help, the temperature logged drops below 15 °C for less time than for those not seeking such advice or medication. However, among those who sought advice from a pharmacist for issues regarding diabetes or feeling down and isolated, the time exposed to 15 °C or less was markedly higher than those who did not (Table 6).

Participants who sought advice from a pharmacist or used casual medicines less than usual during the trial experienced a household temperature below 15 °C for less time than those requiring such services at least as often. The largest difference in time exposure to 18 °C or less occurred for those using casual medicine less frequently than usual (36.5% versus 61.4% among those requiring casual medicines at least as often). 

#### 3.2.2. Access to NHS Services

For most of the participants, supply of the temperature loggers made no difference to their required access to NHS services (all *p* < 0.058, Table 7). For the few participants requiring less interaction with a GP or a practice nurse, requiring fewer home visits, or ending up as a hospital inpatient on fewer occasions, the time exposed to below 15 °C and 18 °C was larger.

Of the nine participants declaring that they had not visited the GP less as a result of the device, only two had recorded a GP visit at all, and all but one had either a respiratory or a cardiovascular illness. 

Six of the 11 respondents declaring no fewer outpatient appointments, as a result of the device, had such an appointment with five of the 11 having respiratory issues and four having some cardiovascular condition. Two participants were admitted as an inpatient through the trial period; one with longstanding cardiovascular problems and the other with respiratory and mental health issues. 

Despite a prior overall degradation in participants’ health in the three months leading up to the trial (Table 1), approximately 20% of participants noted a reduction in their use of pharmacist and NHS services during the trial of the thermometers. This claim is reinforced by the 14 participants stating the temperature logger had helped them over the three-month (*p* = 0.001) period, with a further six respondents being unsure whether the logger had helped or not. 

One participant said it had not helped, claiming that the logger was “inconvenient” and the warning light “annoying”. However, the lone respondent did confess that the badge did raise awareness of the temperature inside the home. 

## 4. Discussion

This exploratory pilot study contributes to the established links between indoor air temperature in homes and health. This is important, because participants with LSIs have potentially poorer outlooks in terms of physical and mental health, which is partly due to sub-optimal living conditions [36,37]. This includes the impact of living in cold homes and associated impact on healthcare services. There was insufficient evidence to show that there were signs that participants felt less cold or less poorly as a result of the trial. However, the study suggested that the wearable temperature loggers improved residents’ awareness (*p* = 0.016) of ambient temperatures and had helped the participants (*p* = 0.001) whilst resulting in a slight reduction in the use of pharmacists and local healthcare services. However, this highlights the need for people with one or more LSIs to maintain higher indoor temperatures for well-being. Given the vulnerability of this population, this must be aligned with improvements to the energy efficiency (e.g., draft proofing, insulation, and heating system upgrades) of their home. While these improvements can lead to a small but positive improvement in health [7], these interventions can have a greater health benefit when targeted at those with a chronic illness [38].

A snapshot of participants’ well-being was also taken prior to the supply of thermometers. In general, well-being had deteriorated across each measured component part (all *p* < 0.05). This picture of deteriorating health was added to participants being more likely to visit a GP than not (*p* = 0.016). However, they were much more likely not to use A & E than require such emergency treatment (*p* < 0.001). This might suggest a cohort routinely seeking help from a doctor to manage their conditions without spilling over into needing emergency treatment.

Residents’ physical performance has been found to be the greatest among those living in homes with a high indoor air temperature [39]. This does not contradict our finding that in households showing a higher proportion of time at which temperatures dip below 15 °C, patients visited the GP surgery more for respiratory, cardiovascular, circulatory, mental health conditions, or cold/flu during the three months prior to the trial. This could be due to homes not having adequate insulation, draught proofing, and heating (i.e., energy efficiency measures). Additionally, this could also stem from participants having a lower awareness of air temperatures within the home. Periods during which internal temperatures dropped below 15 °C were longer for participants less aware of household temperature during the trial. Moreover, participants who managed to maintain temperatures above 18 °C for longer felt less vulnerable, potentially adding to positive mood and benefitting physical health. This finding is consistent with prior recommendations [40]. Suggestions of a need to use heat to create a positive mood align with our findings that the highest recorded temperatures were among participants with severe mental health (20.4 °C, Table 2), perhaps highlighting an association between stress, depression, and the need to keep a house warm.

While the recommended levels of indoor air temperatures for thermal comfort is questionable and based on occupant perceptions, for example, many participant homes fell outside of the safe temperature range [22]. These homes would significantly benefit from household energy efficiency measures to make homes more affordable to heat and improve the indoor thermal comfort. However, these interventions must consider resident behaviours and the provision of both adequate heating and ventilation [8]. These lower temperatures are likely to explain why households declared their health as becoming worse over the study period. It is possible that these households were exposed temporarily to cold homes during the winter months. However, living in poorly heated homes is known to increase the risk of cold related morbidity and mortality [2]. It was not possible to assess the long-term trends in temperature because of the cross-sectional nature of this study. However, these cold and/or poorly insulated homes have been found to suffer from dampness-related contaminants (e.g., mould growth) during the summer months [35]. 

People suffering from conditions exacerbated by winter maintained the temperature of their homes at a slightly higher temperature than those without those conditions. Higher household temperature was also associated with people who had noticed deteriorations in aspects of well-being and LSIs associated with winter over the same three-month period. Such awareness could have been influenced through long-term participation with prior fuel poverty schemes. However, participants revealed a reassurance felt in using the devices, which could lead to longer term feedback in terms of well-being and health.

In an attempt to improve the well-being of the elderly in the UK, the government issued a Winter Fuel Payment (WFP). Despite its intentions, receipt of the WFP was found not to increase indoor temperature or result in better health outcomes [41]. Deploying wearable technology to WFP recipients might lead to their spending it more readily on fuel, ultimately improving their well-being by reducing exposure to excess cold. This approach has the potential to help improve the effectiveness of fuel poverty interventions and avoid potential unintended consequences [9], as well as inform future policies to make fuel poverty and healthcare funding mechanisms more sustainable. 

In this pilot study, we tested the feasibility of using temperature loggers to raise the awareness of being exposed to excessively cold homes. In its current format, this approach provides a useful and cost-effective tool to highlight when indoor temperatures reach a low temperature that can be detrimental to health. However, a more complex and larger study is needed to assess the long-term viability and effectiveness of this public health intervention. Mixed methods approaches should be used in the future to determine typical barriers (e.g., battery life, maintenance) and facilitators (e.g., cost and delivery mechanism in raising awareness) of this approach. This is important to consider because this provides a unique opportunity to influence resident behaviours (by raising awareness) and avoid potential unintended consequences of some prior fuel poverty interventions [9], which can result from a lack of adequate heating and ventilation. If a larger trial proves to be successful, then these must be considered alongside the extensive investment into supporting fuel poor homes with energy efficiency improvements.

This study has a number of limitations. The cross-sectional study design prevented us from assessing the variability of indoor temperatures and impacts on health over a longer period of time. This also prevented us from assessing whether living in cold homes exacerbated or caused ill health and the other health outcomes that were assessed. Furthermore, the small sample size and recruiting participants from a fuel poverty social enterprise may introduce additional bias into the study. Moreover, apart from the temperatures recorded, the information gathered from this study was self-declared by participants without validation from records of service use. We also failed to record whether loggers were worn or used as free-standing devices, which should be addressed in future programs. For this reason, a larger, longer-term study with more objective measures of environmental exposures and health outcomes is needed to increase confidence in the benefits resulting from wearable temperature loggers by patients with an LSI. As indicated above, this should be considered alongside physical built environment interventions that make homes more affordable to heat. This will enable lower-income patients to adequately heat their home and maximise the potential benefits of raising awareness of indoor air temperatures. It may also be necessary to consider other behavioural measures to overcome any potential unintended consequences after undertaking measures such as sealing homes to prevent heat loss. These are necessary to ensure that both ventilation rates and heating patterns maintain good indoor air quality [8,10,13].

Future interventions also need to account for the complexity of interactions between many overlapping built environment and behavioural factors [2]. These include property characteristics (e.g., type, age, energy efficiency, and maintenance levels), household income, situational factors, attitudes, values, and barriers. For example, these interact with the ability of residents to heat the home or access help and/or change heating behaviours [42]. This is important to consider, because the most fuel-poor households may reside in unsafe indoor environments (i.e., cold and damp) despite their risk perception of the potential health risks, use of ventilation, and/or potentially the energy efficiency of the property [43]. Mixed methods approaches may help reveal some of the complexities relating to the built environment, resident behaviours, and health. For example, this could include a qualitative component to assess residents’ barriers and facilitators for different interventions such as the provision of advice. Understanding the interaction between these higher inter-related factors provides an opportunity to intervene and alleviate the health risks associated with cold homes.

Despite the limitations of this study, it demonstrates the potential role for telemetry in the management of LSIs (e.g., reduced dependence on medicines) among the most vulnerable people in society. The improved management of LSIs could lead to the co-benefits of improving the self-management of health conditions and lowering the burden on healthcare systems. This can also contribute to reducing the carbon footprint of the healthcare system. For example, pharmaceuticals makes up 3.5MT CO_2_ (eq.) of the 22.8MT CO_2_ (eq.) emissions generated by England’s NHS, [44] with approximately 1.1 billion items prescribed annually (July 2014–June 2015) [45], and 153 million “over the counter” casual medicines sold during 2016 [46]. 

The need for further investigations into the effectiveness of this intervention is supported by the significant financial resources and carbon footprint needed to manage many chronic diseases [47]. Furthermore, admissions into hospital are more common when patients and their primary carers are unable to manage exacerbations of chronic diseases. 

It is also possible to extend the scope of the wearable temperature logger used in this study to help identify risk factors that could lead to the exacerbation of many LSIs if they go unchecked, thus maximising the potential of other public health prevention activities. Utilising this approach alongside other public health interventions could increase the cost effectiveness of measures to reduce the impact of cold homes [48]; thus further reducing the health inequalities experienced by these vulnerable populations.

## 5. Conclusions

Healthy homes are a pivotal component for the management of an LSI. This study highlights that people with worsening LSIs live in homes that are maintained at higher temperatures than those with less severe illnesses. Maintaining these homes at healthy temperatures which are also well ventilated often requires capital investment and upkeep for policy measures to be sustainable. This highlights the need for more holistic ‘whole system’ approaches that consider the resident behaviours, awareness, and the built environment together. 

The combination of these approaches could help deliver more sustainable public health interventions and provide a cost-effective approach to reducing health inequalities. These could also enable the early discharge of patients from hospital back into their home, delivering co-benefits in reducing the burden on healthcare services and the carbon footprint of healthcare systems. A larger study is needed to fully understand the impact of this intervention and explore the potential for combining with other approaches such as indoor pollutant sensors to reduce the exacerbation of LSIs. 

## Figures and Tables

**Table 1:** Have there been any changes to your health and well-being in the last three months with *p*-values denoting variation from a uniformity of response? Ave: average.

	Much Better	Better	The Same	Slightly Worse	Much Worse
Physical health (*p* = 0.005)	0	1	5	6	10
Ave temp during trial (°C)		18.4	17.1	17.6	19.0
Feeling comfortable (*p* = 0.003)	0	1	9	3	8
Ave temp during trial (°C)		18.4	17.3	18.1	19.2
Feeling anxious (*p* = 0.001)	0	0	9	4	9
Ave temp during trial (°C)			17.4	18.5	18.6
Feeling relaxed (*p* = 0.002)	0	0	9	4	8
Ave temp during trial (°C)			17.5	18.6	18.7
Overall quality of life (*p* = 0.003)	0	0	6	9	7
Ave temp during trial (°C)			17.5	17.8	19.3
Emotional well-being (*p* = 0.002)	0	0	6	10	6
Ave temp during trial (°C)			17.4	17.9	19.3

**Table 2:** Which health problems have you experienced in the last three months with *p*-values relative to the chance of a health problem versus not?

	No	Mild	Moderate	Severe
Respiratory (*p* = 0.513)	9	2	0	10
Ave temp during trial (°C)	17.5	17.7		18.3
Cardiovascular (*p* = 0.513)	9	6	1	5
Ave temp during trial (°C)	17.1	18.2	18.8	18.5
Circulatory (*p* = 0.275)	8	5	4	4
Ave temp during trial (°C)	17.1	18.2	18.2	18.7
Mental health (*p* = 0.074)	14	1	1	4
Ave temp during trial (°C)	17.5	18.8	18.1	20.4
Cold/Flu (*p* = 0.003)	16	0	2	1
Ave temp during trial (°C)	17.6		17.8	18.1
Anxiety (*p* = 0.491)	11	2	2	4
Ave temp during trial (°C)	17.5	18.7	17.3	
Learning difficulties (*p* < 0.001)	18	0	0	1
Ave temp during trial (°C)	17.7			

**Table 3:** Since receiving assistance, have there been any changes to your health and well-being with *p*-values denoting the difference between improvement or worsening (ignoring those feeling the same)?

	Much Better	Better	The Same	Slightly Worse	Much Worse
Physical health (*p* = 0.763)	2	3	10	4	2
Ave temp during trial (°C)	18.3	17.8	18.1	18.1	17.2
Feeling comfortable (*p* = 0.366)	2	5	10	2	2
Ave temp during trial (°C)	18.3	17.4	18.2	18.1	18.8
Feeling anxious (*p* = 0.739)	3	2	12	3	1
Ave temp during trial (°C)	18.1	17.6	18.1	18.1	
Feeling relaxed (*p* = 1.000)	3	2	11	4	1
Ave temp during trial (°C)	18.1	17.6	18.0	18.4	
Overall quality of life (*p* = 0.739)	3	2	12	1	3
Ave temp during trial (°C)	18.1	17.6	18.0	18.8	18.1
Emotional wellbeing (*p* = 0.739)	3	2	12	3	1
Ave temp during trial (°C)	18.1	17.6	18.0	18.3	

**Table 4:** Have you used the following services in the last three months for any respiratory, cardiovascular, circulatory, mental health conditions, or cold/flu (*p*-values representing any visits versus none)? GP: general practitioner.

	0	1 to 3	4 to 6	7+	Total	Ave
Interval mean	0	2	5	8		
GP appointment (*p* = 0.016)	5	14	2	0	38	1.7
Ave temp during trial (°C)	18.8	17.7	17.7			
Time exposed to 15 °C or less	11.1%	17.2%	23.1%			
Time exposed to 18 °C or less	47.7%	61.2%	55.9%			
Hospital appointment (*p* = 0.275)	8	9	2	2	44	2.0
Ave temp during trial (°C)	18.1	18.0	17.9	18.1		
Time exposed to 15 °C or less	19.1%	14.5%	8.7%	13.65%		
Time exposed to 18 °C or less	55.6%	56.1%	62.9%	57.428%		
Accident & Emergency Department (A & E, *p* < 0.001)	20	1	0	0	2	0.1
Ave temp during trial (°C)	18.1	17.2				
Time exposed to 15 °C or less	15.9%	17.5%				
Time exposed to 18 °C or less	55.5%	74.6%				

**Table 5:** Badge and monitor feedback section 1—well-being versus last winter and average temperature (with *p*-values representing positive versus negative responses).

	Positive	Neutral	Negative
Have you been more aware of the temperature of your house this winter? (*p* = 0.016)	16		5
Ave temp during trial (°C)	18.2		17.5
Time exposed to 15 °C or less	14.1%		24.7%
Time exposed to 18 °C or less	55.3%		61.7%
Have you felt less vulnerable or down this winter? (*p* = 0.071)	11	6	4
Ave temp during trial (°C)	18.6	17.3	17.6
Time exposed to 15 °C or less	11.4%	28.6%	14.5%
Time exposed to 18 °C or less	49.4%	62.8%	66.9%
Have you felt as cold this winter? (*p* = 0.808)	8	4	9
Ave temp during trial (°C)	17.9	17.8	18.2
Time exposed to 15 °C or less	17.2%	22.5%	13.1%
Time exposed to 18 °C or less	56.2%	56.6%	56.5%
Have you been feeling poorly this winter; how does it compare to last year? (*p* = 0.564)	5	9	7
Ave temp during trial (°C)	18.0	18.1	18.0
Time exposed to 15 °C or less	14.9%	19.8%	12.4%
Time exposed to 18 °C or less	58.6%	54.8%	56.3%

**Table 6:** Temperature logger feedback section 2—pharmacy and casual medicines.

	Casual Medicines	N/a	Pharmacist
Breathing, coughing, colds, and sore throats	3		7
Ave temp during trial (°C)	17.8	18.1	18.1
Time exposed to 15 °C or less	10.1%	19.8%	12.7%
Time exposed to 18 °C or less	66.2%	53.7%	56.8%
Skin irritations	0		3
Ave temp during trial (°C)		18.0	18.5
Time exposed to 15 °C or less		17.5%	9.1%
Time exposed to 18 °C or less		57.7%	50.5%
Diarrhoea and vomiting	1		2
Ave temp during trial (°C)	17.9	18.0	18.7
Time exposed to 15 °C or less	8.7%	18.0%	6.2%
Time exposed to 18 °C or less	62.9%	58.1%	41.9%
Flu	2		3
Ave temp during trial (°C)	17.8	18.1	17.7
Time exposed to 15 °C or less	10.1%	16.9%	15.6%
Time exposed to 18 °C or less	66.2%	53.8%	66.0%
Heart and circulation	0		9
Ave temp during trial (°C)		18.1	18.0
Time exposed to 15 °C or less		17.3%	14.4%
Time exposed to 18 °C or less		57.5%	55.1%
Diabetes	0		3
Ave temp during trial (°C)		18.1	17.7
Time exposed to 15 °C or less		15.1%	23.1%
Time exposed to 18 °C or less		56.5%	55.9%
Pain management, joint problems, and muscle problems	1		6
Ave temp during trial (°C)	17.6	18.1	17.9
Time exposed to 15 °C or less	11.4%	16.3%	16.2%
Time exposed to 18 °C or less	69.4%	55.8%	54.9%
Feeling down or isolated	0		3
Ave temp during trial (°C)		18.1	17.2
Time exposed to 15 °C or less		14.6%	28.6%
Time exposed to 18 °C or less		55.8%	62.2%

**Table 7:** Since the badge was fitted, are you using the following services less than usual? Here, *p*-values representing difference between positive and negative responses.

	No	Yes
Pharmacist (*p* = 0.058)	8	2
Ave temp during trial (°C)	17.9	18.4
Time exposed to 15 °C or less	14.0%	7.1%
Time exposed to 18 °C or less	59.9%	53.9%
Casual Medicines (*p* = 0.058)	8	2
Ave temp during trial (°C)	17.9	18.8
Time exposed to 15 °C or less	14.3%	4.9%
Time exposed to 18 °C or less	61.4%	36.5%
Prescription Medicine (*p* = 0.007)	10	1
Ave temp during trial (°C)	17.8	
Time exposed to 15 °C or less	16.0%	
Time exposed to 18 °C or less	58.8%	
GP/Nurse/ Home Visit (*p* = 0.035)	9	2
Ave temp during trial (°C)	18.1	17.2
Time exposed to 15 °C or less	12.9%	17.5%
Time exposed to 18 °C or less	55.2%	74.6%
Outpatient (*p* = 0.013)	11	2
Ave temp during trial (°C)	17.9	17.6
Time exposed to 15 °C or less	15.5%	12.9%
Time exposed to 18 °C or less	57.6%	68.7%
Inpatient (*p* = 0.021)	10	2
Ave temp during trial (°C)	17.9	17.2
Time exposed to 15 °C or less	15.9%	17.5%
Time exposed to 18 °C or less	56.3%	74.6%

## References

[1] Orchard C. (2015). Adult Health in Great Britain 2013. Opinions and Lifestyle Survey. https://www.ons.gov.uk/peoplepopulationandcommunity/healthandsocialcare/healthandlifeexpectancies/compendium/opinionsandlifestylesurvey/2015-03-19/adulthealthingreatbritain2013#self-reported-long-standing-illness-or-disability.

[2] Sharpe R.A., Taylor T., Fleming L.E., Morrissey K., Morris G., Wigglesworth R. (2018). Making the case for ‘whole system’ approaches: Integrating public health and housing. Int. J. Environ. Res. Public Health.

[3] Tanner L.M., Moffatt S., Milne E.M., Mills S.D., White M. (2013). Socioeconomic and behavioural risk factors for adverse winter health and social outcomes in economically developed countries: A systematic review of quantitative observational studies. J. Epidemiol. Community Health..

[4] Liddell C., Morris C. (2010). Fuel poverty and human health: A review of recent evidence. Energy Policy.

[5] De Vries R., Blane D. (2013). Fuel poverty and the health of older people: The role of local climate. J. Public Health. (Oxf)..

[6] Wilkinson P., Pattenden S., Armstrong B., Fletcher A., Kovats R.S., Mangtani P., McMichael A.J. (2004). Vulnerability to winter mortality in elderly people in Britain: Population based study. BMJ.

[7] Maidment C.D., Jones C.R., Webb T.L., Hathway E.A., Gilbertson J.M. (2014). The impact of household energy efficiency measures on health: A meta-analysis. Energy Policy.

[8] Sharpe R.A., Thornton C.R., Nikolaou V., Osborne N.J. (2015). Higher energy efficient homes are associated with increased risk of doctor diagnosed asthma in a UK sub population. Environ. Int..

[9] Shrubsole C., Macmillan A., Davies M., May N. (2014). 100 Unintended consequences of policies to improve the energy efficiency of the UK housing stock. Indoor Built Environ..

[10] Sharpe T., Farren P., Howieson S., Tuohy P., McQuillan J. (2015). Occupant Interactions and Effectiveness of Natural Ventilation Strategies in Contemporary New Housing in Scotland, UK. Int. J. Environ. Res. Public Health..

[11] Mudarri D., Fisk W.J. (2007). Public health and economic impact of dampness and mold. Indoor Air.

[12] Nicol S., Roys M., Garrett H. (2015). The Cost of Poor Housing to the NHS.

[13] Sharpe R.A., Machray K.E., Fleming L.E. (2018). Modelling the Impact of Fuel Poverty and Energy Efficiency on Health.

[14] Bennett M. (2018). Excess Winter Mortality in England and Wales: 2017 to 2018 (Provisional) and 2016 to 2017 (Final). Excess Winter Mortality in England and Wales. https://www.ons.gov.uk/peoplepopulationandcommunity/birthsdeathsandmarriages/deaths/bulletins/excesswintermortalityinenglandandwales/2017to2018provisionaland2016to2017final.

[15] Kmietowicz Z. (2018). Winter pressure: Government responses across UK. BMJ Br. Med. J..

[16] Healy J. (2003). Excess winter mortality in Europe: A cross country analysis identifying key risk factors. J. Epidemiol. Community Health.

[17] Bouzarovski S., Tirado Herrero S. (2017). The energy divide: Integrating energy transitions, regional inequalities and poverty trends in the European Union. Eur. Urban Reg. Stud..

[18] Thomson H., Snell C., Bouzarovski S. (2017). Health, Well-Being and Energy Poverty in Europe: A Comparative Study of 32 European Countries. Int. J. Environ. Res. Public Health.

[19] PHE (2019). Wider Determinants of Health. Natural and Built Environment. https://fingertips.phe.org.uk/profile/wider-determinants/data#page/0/gid/1938133043/pat/6/par/E12000009/ati/102/are/E06000052.

[20] O’Sullivan K., Barnard L.T., Viggers H., Howden-Chapman P. (2016). Child and youth fuel poverty: Assessing the known and unknown. People Place Policy.

[21] Hajat S., Kovats R.S., Lachowycz K. (2007). Heat-related and cold-related deaths in England and Wales: Who is at risk?. Occup. Environ. Med..

[22] Sharpe R.A., Thornton C.R., Osborne N.J. (2014). Modifiable Factors Governing Indoor Fungal Diversity and Risk of Asthma. Clin. Exp. Allergy.

[23] O’Sullivan K.C., Howden-Chapman P.L., Fougere G.M. (2015). Fuel poverty, policy, and equity in New Zealand: The promise of prepayment metering. Energy Res. Soc. Sci..

[24] BSI (2011). Code of Practice for Control of Condensation in Buildings.

[25] Bai L., Li Q., Wang J., Lavigne E., Gasparrini A., Copes R., Chen H. (2018). Increased coronory heart disease and stroke hospitalisations from ambient temperatures in Ontario. BMJ Heart.

[26] Gilbertson J., Grimsley M., Green G. (2012). Psychosocial routes from housing investment to health: Evidence frtom England’s home energy efficiency scheme. Energy Policy.

[27] Dixon N. (2017). Guide to Managing Ethical Issues in Qualityimprovement or Clinical Audit Projects. https://www.hqip.org.uk/wp-content/uploads/2017/02/guide-to-managing-ethical-issues-in-quality-improvement-or-clinical-audit-projects.pdf.

[28] Court C., Sharpe R.A., Cohen R.L., Howarth L., Puttock G., Wigglesworth R. (2017). Health Starts Where We Live. https://www.cornwall.gov.uk/media/29038760/public-health-annual-report-2017_web.pdf.

[29] Roberts D., Vera-Toscano E., Phimister E. (2015). Fuel poverty in the UK: Is there a difference between rural and urban areas?. Energy Policy.

[30] Bouzarovski S., Cauvain J. (2016). Spaces of exception: Governing fuel poverty in England’s multiple occupancy housing sector. Space Polity.

[31] Hills J. (2011). Fuel Poverty: The Problem and its Measurement.

[32] Hills J. (2012). Getting the Measure of Fuel Poverty: Final Report of the Fuel Poverty Review.

[33] Moore R. (2012). Improving the Hills Approach to Measuring Fuel Poverty.

[34] Moore R., Wilkinson B., Jobson K. (2017). An Assessment Tool for Low Income/High Costs (LIHC) Fuel Poverty—Final Stage 3.

[35] Sharpe R.A., Le Cocq K., Nikolaou V., Osborne N.J., Thornton C.R. (2016). Identifying risk factors for exposure to culturable allergenic moulds in energy efficient homes by using highly specific monoclonal antibodies. Environ. Res..

[36] Liddell C., Guiney C. (2015). Living in a cold and damp home: Frameworks for understanding impacts on mental well-being. Public Health.

[37] Shiue I. (2016). Cold homes are associated with poor biomarkers and less blood pressure check-up: English Longitudinal Study of Ageing. Environ. Sci. Pollut. Res..

[38] Thomson H., Thomas S., Sellstrom E., Petticrew M. (2013). Housing Improvements for Health and Associated Socio-economic Outcomes. Cochrane Database of Systematic Reviews. http://onlinelibrary.wiley.com/doi/10.1002/14651858.CD008657.pub2/abstract.

[39] Lindemann U., Oksa J., Skelton D.A., Beyer N., Klenk J., Zscheile J., Becker C. (2014). Effect of cold indoor environment on physical performance of older women living in the community. Age Ageing.

[40] Jevons R., Carmichael C., Crossley A., Bone A. (2016). Minimum indoor temperature threshold recommendations for English homes in winter—A systematic review. Public Health.

[41] Angelini V., Daly M., Moro M. (2019). Public Health Research. The Effect of the Winter Fuel Payment on Household Temperature and Health: A Regression Discontinuity Design Study.

[42] Tod A.M., Lusambili A., Homer C., Abbott J., Cooke J.M., Stocks A.J., McDaid K.A. (2012). Understanding factors influencing vulnerable older people keeping warm and well in winter: A qualitative study using social marketing techniques. BMJ Open.

[43] Sharpe R.A., Thornton C.R., Nikolaou V., Osborne N.J. (2015). Fuel poverty increases risk of mould contamination, regardless of adult risk perception & ventilation in social housing properties. Environ. Int..

[44] Sustainable Development Unit (2015). Carbon Footprint update for NHS in England. https://www.sduhealth.org.uk/policy-strategy/reporting/nhs-carbon-footprint.aspx.

[45] McAuley C. (2015). Table of Prescription. https://www.nhsbsa.nhs.uk/prescription-data/prescribing-data/prescription-volume-and-prescription-cost.

[46] Buckley S. (2017). Data on Over the Counter Medicines. https://www.nhsbsa.nhs.uk/prescription-data/prescribing-data/data-over-counter-medicines.

[47] Mukherjee M., Stoddart A., Gupta R.P., Nwaru B.I., Farr A., Heaven M., Lyons R.A. (2016). The epidemiology, healthcare and societal burden and costs of asthma in the UK and its member nations: Analyses of standalone and linked national databases. BMC Med..

[48] Owen L., Fischer A. (2019). The cost-effectiveness of public health interventions examined by the National Institute for Health and Care Excellence from 2005 to 2018. Public Health.

